# Uterine Artery Embolization: A Case Report on Primary Postpartum Hemorrhage Management

**DOI:** 10.7759/cureus.63956

**Published:** 2024-07-06

**Authors:** Shruti H Mandviya, Snehlata Hingway, Vishal Ghule

**Affiliations:** 1 Pathology and Laboratory Medicine, School of Allied Health Sciences, Datta Meghe Institute of Higher Education and Research, Wardha, IND; 2 Pathology, Jawaharlal Nehru Medical College, Datta Meghe Institute of Higher Education and Research, Wardha, IND; 3 Pathology, School of Allied Health Sciences, Datta Meghe Institute of Higher Education and Research, Wardha, IND

**Keywords:** fertility, interventional radiology, catheters, postpartum hemorrhage (pph), uterine artery embolization

## Abstract

When traditional therapies fail to control obstetric emergencies such as postpartum hemorrhage (PPH), uterine artery embolization (UAE) has become an essential intervention. This case study demonstrates the effective use of UAE in a 32-year-old patient who had an elective cesarean section and was experiencing refractory PPH. Despite initial attempts at controlling bleeding with uterotonic agents and surgical intervention, the hemorrhage persisted, necessitating packed red blood cell transfusion. A multidisciplinary team opted for UAE due to the patient's deteriorating condition. Gelatin sponge particles were utilized as embolic agents, resulting in the immediate cessation of uterine blood flow and the stabilization of the patient. This case underscores the importance of early detection, collaborative decision-making, and prompt intervention in managing PPH. UAE offers several advantages, including targeted vascular occlusion, rapid bleeding control, and the preservation of fertility. Further research and practice are warranted to optimize UAE techniques and enhance outcomes in obstetric emergencies. The primary cause of severe maternal morbidity and death is postpartum hemorrhage. For successful uterine artery embolization (UAE), prompt management is essential. UAE is widely acknowledged as a dependable and safe process.

## Introduction

For the past two decades, interventional radiologists have employed uterine artery embolization (UAE) as a treatment for various obstetric and gynecological conditions characterized by severe, uncontrollable bleeding. This procedure typically entails selectively embolizing one of the two uterine arteries to halt the bleeding. During the procedure, the patient may receive either general anesthesia or intravenous analgesia, with the former being administered by an anesthesiologist [1]. In certain cases, the patient can be treated in the radiology suite while under observation by the radiology staff if conscious. A special catheter is inserted via a femoral artery, which is located in the upper thigh, and then advanced through the internal iliac artery. Next, the catheter is navigated to the uterine artery, a branch of the anterior division of the internal iliac artery that supplies blood to the uterus. If necessary, further catheterization may involve navigating into the ovarian arteries, particularly when assessing or targeting collateral circulation to the uterus is required. This procedure can be performed using a single femoral artery puncture, with the catheter being directed through the lower aorta and into the opposing internal iliac artery [2].

Many radiologists prefer to use both sides of a catheter in the femoral arteries to access the adjacent uterine artery and reduce the associated ovarian dose. Uterine artery embolization (UAE) has a high success rate and is minimally invasive, making it the preferred treatment for postpartum hemorrhage (PPH). Although UAE has a great success rate in treating PPH, patients with uncontrollable PPH may require surgery to stop the bleeding. Postpartum hemorrhage remains one of the leading causes of maternal death, and delays in diagnosis can lead to delayed treatment, which can have detrimental effects on the morbidity and mortality of mothers. To prevent major bleeding in many situations, several protocols have been created, and primary postpartum hemorrhage is typically diagnosed and treated as soon as possible [3,4].

## Case presentation

This case report highlights a 32-year-old female in her second pregnancy, who had one prior childbirth and underwent an elective cesarean section at 39 weeks due to breech presentation. After giving birth to a healthy 3.4 kg male infant, the patient began to experience excessive bleeding, totaling over 1500 mL, which indicated a potential postpartum hemorrhage (PPH). To address the bleeding, the physician performed a uterine massage and administered uterotonic agents, such as methylergonovine and oxytocin. However, these interventions were unsuccessful in stopping the bleeding, necessitating further investigation. Despite ongoing hemorrhage, the physician performed an exploratory laparotomy. During the surgical procedure, no apparent source of bleeding was identified, and the uterus was found to be atonic but intact. In an attempt to control the bleeding, the physician performed a bilateral ligation of the uterine arteries, but the bleeding continued unchecked, prompting the patient to receive a packed red blood cell transfusion to maintain stable hemodynamics.

**Figure 1:**
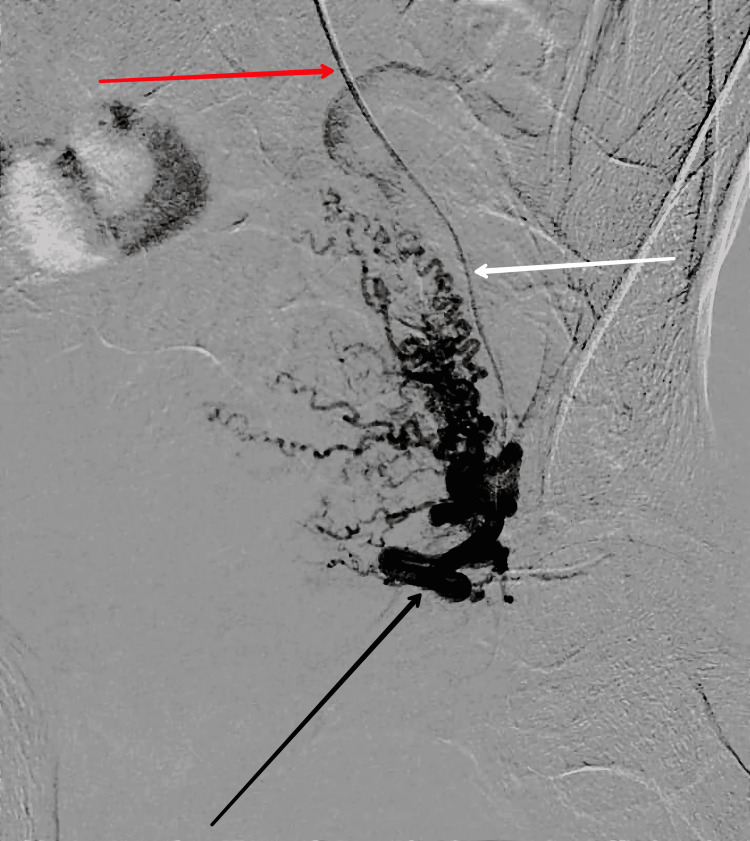
Pre-shoot before the embolization of the uterine artery. The red arrow shows the main catheter; the white arrow shows the microcatheter; the black arrow shows the feeder artery.

A multidisciplinary team decided to use uterine artery embolization (UAE), as the patient's condition was deteriorating and the bleeding was persistent. In this procedure, gelatin sponge particles were utilized as embolic agents and were inserted into the uterine arteries under fluoroscopic guidance. After the procedure, immediate post-procedural angiography confirmed that the uterine arteries had been successfully blocked, stopping blood flow to the uterus. This therapeutic approach proved to be life-saving in the management of refractory postpartum hemorrhage, and it highlights the importance of prompt action in addressing obstetric emergencies that are unresponsive to conventional therapies. It also emphasizes the significance of interdisciplinary collaboration and timely intervention strategies, as this procedure is not considered major surgery. A pre-embolization shot is taken before the embolization, as shown in Figure 1.

**Figure 2:**
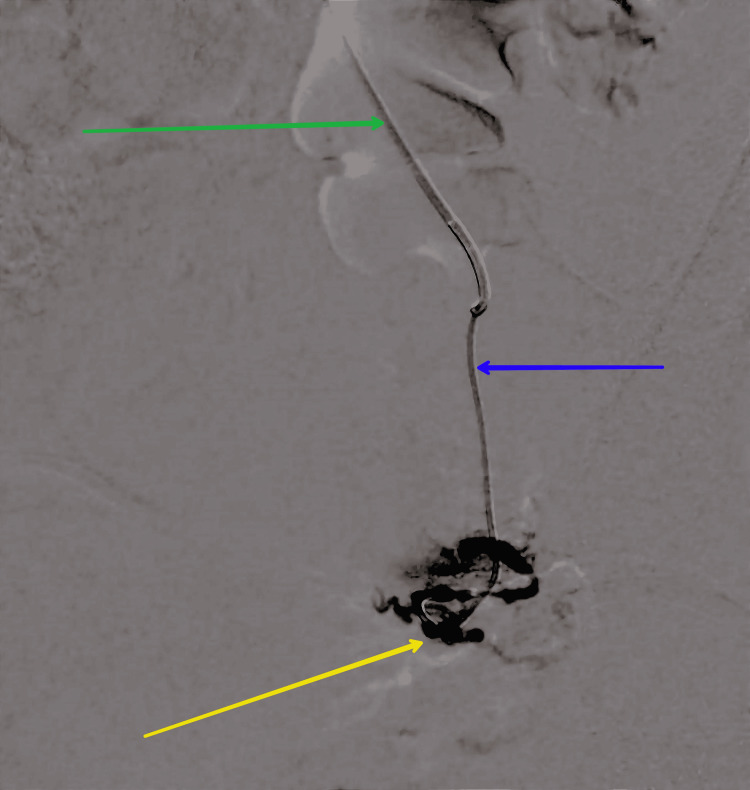
Post-shoot showing the complete obliteration of feeder artery embolization done. The green arrow shows the main catheter; the blue arrow shows the microcatheter; the yellow arrow shows the complete obliteration of the feeder artery.

The primary objective of treating primary postpartum hemorrhage (PPH) is to stabilize and resuscitate the patient by establishing intravenous access, administering crystalloid fluids, and closely monitoring hemodynamic parameters. After the patient is moved to the interventional radiology suite for the uterine artery embolization (UAE) procedure, as shown in Figure 2, catheters are inserted into the uterine arteries using fluoroscopic guidance. Embolic agents, typically made of coils or particulate matter, are then inserted into the uterine arteries to cause vascular occlusion and arrest uterine bleeding. Uterine artery embolization (UAE) is a minimally invasive procedure designed to manage postpartum hemorrhage by obstructing the blood supply to the uterus. This technique involves the insertion of a catheter into the uterine arteries, through which embolic agents are introduced to block the blood flow. By reducing the blood supply, UAE effectively controls excessive bleeding and can be a valuable intervention in cases where traditional surgical methods are not feasible or have failed.

## Discussion

Primary postpartum hemorrhage (PPH) is a significant contributor to maternal morbidity and mortality worldwide, despite advancements in obstetric care. Refractory cases of PPH pose significant challenges and often require nonconventional interventions. Effective post-procedural care involves monitoring vital signs, surveillance for procedural complications, and administering appropriate analgesics and anti-inflammatory agents. Allergic reactions or contrast-induced nephropathy must also be diligently monitored. Uterine artery embolization (UAE) and selective uterine artery embolization (SUAE) are minimally invasive interventions used to achieve hemostasis in cases of primary postpartum hemorrhage when conservative and surgical interventions prove inadequate. These techniques involve deliberate blood flow obstruction to the uterus, in either a generalized or a targeted manner, to mitigate hemorrhagic sequelae. Uterine artery embolization (UAE) has become an important adjuvant therapy for treating PPH, especially when more conservative measures fail to control bleeding [5].

Considering the urgency of primary postpartum hemorrhage as a medical emergency, obstetricians and interventional radiologists should take into account the findings presented here when determining the optimal course of action to increase the success rate of UAE [6]. The effectiveness of uterine artery embolization lies in its ability to induce targeted vascular occlusion, which decreases uterine blood flow and promotes hemostasis. This procedure achieves its objective by selectively obliterating the uterine arteries, thus limiting the amount of blood reaching the uterus while preserving blood flow to surrounding organs [7].

The numerous benefits of minimally invasive approaches include quick bleeding control, fertility preservation, and avoiding time-consuming surgical procedures. In a case where uterotonic agents and surgical interventions were unsuccessful, UAE demonstrated effectiveness in achieving hemostasis and stabilizing the patient's clinical status [8]. To prevent additional maternal morbidity and mortality, the early detection of refractory hemorrhage and the prompt initiation of UAE are crucial. Uterine artery embolization is a beneficial tool in the multidisciplinary management of refractory postpartum hemorrhage. Further research and practice are necessary to improve patient outcomes, refine techniques, and deepen our understanding of UAE's features in PPH management [9]. Direct routes of infection spread include the endometrial cavity, adnexa, bowel, or blood during embolization. Pyomyoma, which presents with a classic triad of pain in the pelvis, sepsis, and no known source of infection, can be challenging to diagnose due to its similarity to post-embolization syndrome (PES), which may also present with fever, elevated white cell count, and pelvic pain within a week of UAE [10].

Follow-up

Routine gynecological assessments include the following: 1) scheduled gynecological evaluations to monitor uterine health and 2) ultrasound examinations to assess the integrity of the uterine arteries and ensure no recurrence of bleeding.

Patient education and support include the following: 1) education on recognizing early signs of potential complications such as delayed bleeding or infection and 2) psychological support and counseling to address any emotional or mental health concerns following the traumatic event of PPH and subsequent interventions.

## Conclusions

This case report underscores the critical role of uterine artery embolization (UAE) in the management of refractory postpartum hemorrhage (PPH) when conventional therapies such as uterotonic agents and surgical interventions fail. The successful use of UAE in the presented case highlights its effectiveness in achieving rapid hemostasis, stabilizing the patient, and preserving fertility. Early detection, prompt decision-making, and a multidisciplinary approach are paramount in managing PPH to reduce maternal morbidity and mortality. UAE, as a minimally invasive procedure, offers significant benefits, including targeted vascular occlusion and quick bleeding control. Further research and continued practice will enhance UAE techniques and outcomes in obstetric emergencies, reinforcing its vital role in contemporary obstetric care. Uterine artery embolization is a safe and effective method for managing primary postpartum hemorrhage. Future research and practice are necessary to refine UAE techniques and deepen our comprehension of their effectiveness in treating PPH. By fostering collaboration between obstetricians and interventional radiologists, we can enhance UAE's integration into the multidisciplinary treatment of refractory postpartum hemorrhage, ultimately leading to better maternal health outcomes, reduced morbidity, and lower mortality rates.
